# Supplementary material for: An emerging TIMER-2C framework for addressing barriers to research culture and productivity among local healthcare providers in the Middle East and sub-Saharan Africa: A qualitative study and modified Delphi approach
Source: PLOS Glob Public Health. 2026 Apr 8;6(4):e0006046. doi: 10.1371/journal.pgph.0006046 (PMC13061179; doi:10.1371/journal.pgph.0006046)
Supplement: S1 Appendix — (DOCX) [file pgph.0006046.s002.docx]

**S1 Appendix: SAMPLE CODES AND EXEMPLARY QUOTES BY THEME**

| **TIME** |  |
| --- | --- |
| Lack of Time/ fatigue | There are huge teaching loads, universities are not sufficiently funded to allow time for research for the faculty. So, they teach a lot of courses. and that of course means that there is no room for research over there or very, very limited research outside” [MENA senior male researcher]  “that part of the world it is just crazy. In that context, I was saying that research falls at the bottom of the priorities unfortunately, research in the structured way that we were talking about.” [MENA senior female researcher]  “We are overwhelmed... so you are really just barely surviving, just trying to stay from drowning in the service delivery work... You are actually overwhelmed… I would have burnt out completely if I had not left… it wasn’t humanly sustainable.” [SSA senior male researcher] |
| Sustainability/ continuity | “mentored research is you know...something that is very important for the continuity, sustainability of research culture in the institutions.” [SSA senior female researcher]  “I have discovered the capacity to take people and developing that kind of expertise, the need to cascade them in order to have along the generation. So it’s train the next trainer approach… the mentees become the mentors… each student should give back to the next.” [SSA senior female researcher]  “It’s a mentorship thing. You are not going to grow good researcher in a day.  Research is a commitment for a period of time,, mentorship-attitude, mentorship-growth. encouraging people to truly mentor and build the next generation.” [SSA senior female researcher] |
| Time and competing interests | “There is another barrier, they cannot just leave there their families and go for a PhD somewhere else. The local PhD programs which are not the best, these are very new… I have an arrangement with the London school of hygiene and tropical medicine.” [MENA senior female researcher]  “When I come home, I can do some research work or play with my children but for a woman, the same responsibilities plus running the family... It will be a burden on the woman” [SSA senior male researcher]  “I was busy with clinical activities. I didn’t have time for research and also there was no opportunity.” [[SSA senior male researcher] |

| **INSTABILITY/ INTEREST/ INFRASTRUCTURE** |  |
| --- | --- |
| Building research circle | “Surrounding yourself with the right people, I call it the circle of safety. Unfortunately, we tend to find ourselves with the wrong people, and the apathy of the wrong people would drive your love for this work down.” [SSA senior female researcher] |
| Lacks importance | “Culture around the research or lack of an appreciation for the utility of research.” [SSA senior female researcher]  “This is sort of a common understanding that these are things that you would not much need as a physician… it is a common culture. This is something that you are just doing for grades, you pass that year... we don’t see the value of it, we don’t use it, we don’t apply it as much.” [MENA junior male researcher] |
| Desire to make a difference | “I wanted to quantify the problem, so research was at the back of my mind... questions around how we provide services, how we organize the services production,… how we can really be truly responsive to people’s needs. I wasn’t equipped.. so, I trained…” [SSA senior male researcher] |
| Administration & compliance | “There is a certain level of research administration that people need to learn and they don’t understand… what it takes to write to an IRB. You can have a scientist who knows how to do the science and doesn’t know how to manage administration around it, that is not a good PI.” [SSA senior female researcher]  “The complexities of the IRBs is quite significant and extremely valuable in the Middle East.” [MENA senior female researcher] |
| Innovation/ nurture creativity | “This system was set up very much on colonial systems, the colonialists themselves have changed theirs, they have evolved and we are still sitting there. We need to change that.” [SSA senior male researcher]  “Unless you allow people to be curious, they can’t be researchers even when they have great ideas they just can’t be, because you have destroyed their innovation.” [SSA senior female researcher] |
| Institutional/ Infrastructure/ DON'T CARE | “I tried different opportunities to come back… many universities did not even consider my application... your career path is a researcher what we need are teachers.” [MENA senior male researcher] |
| Local agenda and ownership of research | “If we had our own funding for health systems and our health programs, we would have our own target. We would be less at the disadvantage in determining the direction of our health programs.” [SSA senior female researcher] |
| Passion and resilience | “So you have to find local solutions. None of my publications has received any funding: Zero funding, and we used the resource which is commonly available to everyone. Where is the resource? In between your two ears – out knowledge.” [ASIA senior male researcher] |
| Institutional policies | “We have a lot of good policies that we do as a country, like there is a national research ethnics group... We have a database where if you want a part do research in a province better get a proposal this province give you feedback, they give you all of that. So that system is nice.” [SSA senior female researcher] |
| Self-made/ initiative | “At a certain point, you have to make a concerted effort to improve it. My writing has improved, because you read, you observe, you change but for some people who are stuck in that.” [SSA senior female researcher]  “So you have to find local solutions. I tell everyone we talk about funding, none of my publication has received any funding: Zero funding, and we used the resource which is commonly available to everyone. Where is the resource? In between your two ears.” [ASIA senior male researcher]  “There could be very good mentors that could be very good sponsors but if you yourself are not driven, and don’t take advantage of that mentorship and [ship… you may become a researcher but that’s not the ideal situation or you might not get to the potential that you might want to get to.” [SSA senior female researcher] |
| Resistance and negativity | “There is bureaucracy that you have to overcome, something you are supposed to do for five years, you spend four years overcoming bureaucracy and then by time you are finally able to train the person… you are able to do the research but can’t do it as you wanted.” [ASIA senior male researcher]  “At institutional levels, we have already discussed. They have to support, they have to encourage, they have to record, they have to give protected time like every paper has been typed by me, I don't have a secretary… It takes a long time...I could have done more thinking but still I have to do all this.” [ASIA senior male researcher] |

| **MEANS OF FUNDING** |  |
| --- | --- |
| Corruption/ Accountability | “There was funding allocated for students to do their field work…and do the data collection, every student got some money to do that… We have been hearing different stories that there is corruption, money goes somewhere else unfortunately.” [[MENA junior male researcher] |
| Finance/ funding | “There is no connect to people … even on a political level and so there is no money for it… people running research, but they don’t have money. You need money for research and so unfortunately until governments start funding this work, it hard.” [SSA senior female researcher]  “… has quite enlightened leadership... They decided to invest in science and in education, in fact I work here, and it is called education city. Literally it is a city for education science and research… Fortunately we have good access to funding… I am basically enjoying my career… We have had really extremely high productivity; I can tell you for sure if I was in the USA I could not have had this kind of success.” [MENA senior male researcher] |
| Incentives/Awards | “For each publication, you will get some incentive… based on the number of publications you have… so you will be recognized… you will also get some financial incentive and also get some promotion based on that.” [MENA junior female researcher] |
| Job satisfaction/ brain drain | “People that are trained are not into the system they are outside of the country, brain drain and so on, which affects research.” [MENA senior male researcher] |
| Purity of science/ Tied Aid | “The thing is that they also come with their agendas. Agenda is not necessarily a bad thing but they also come with their agendas... Again there are others have had foundation grants where honestly they have no agenda except we have to fund you research” [SSA senior male researcher] |
| Resourcefulness | “Now when science is multidisciplinary and collaborative, we struggle because we are so used to despising the other one, "I am the best in this, I am the best in that" but now you have to work with everybody because it takes a whole village to do a clinical trial.” [SSA senior female researcher]  “African countries should not think of PEPFAR (The President's Emergency Plan for AIDS Relief)...the end of PEPFAR is inevitable" … We need to be funding ourselves but the same way we are dealing with research is the same way we are dealing with funding, we have not moved, we have not invested in the research.” [SSA senior female researcher]  “Encourage them, it could even be co-funding by the way… if someone for example would apply and they get some kind of funding we match it, something that is pretty common in the united states… So in this way also we support their successes.” [MENA senior male researcher] |
| Scholarship support | “… a lot of that was fueled by the research money and then of course Fogarty program, nearly anyone who is anyone in Africa who is doing HIV work has the Fogarty training.” [SSA senior male researcher]  “…reflecting on my very own experience. I don’t think I would have gotten to the place where I am at now without the scholarship, honestly… part of the challenge is having the idea even before giving someone the scholarship. The selection criteria of the students should be students who represent institutions so that building that capacity of that individual ends in building the capacity of the institution that the individual represents. I very much applaud the Kenyan experience with University of Washington… everyone who comes to have a masters or a PhD, they have already established positions. They finish their degree and then they go back and they are involved in teaching, training of trainers and all of that. That I think is the model to choose. In Sudan for example, the sad reality is... the majority just applied as individuals and they qualified as individuals, they have good potential skills and all of that but not so much connection to an institution that they would invest in building.” [MENA junior male researcher] |

| **EXPERTISE & EXPERIENCE** | Quote |
| --- | --- |
| Parachute science/ Extractive practices | “Many of the international collaborations that actually happened in our region, it was really more they needed partners locally... It involves some capacity building but at the end of the day, really the research is American research or European research... I won’t say that it will necessarily build sufficient capacity. I prefer honestly the more indigenous, where the research is led by people here on the ground, they build their own career... The condition for international collaborations here, two thirds of the funds have to be spent locally...” [MENA senior male researcher]  “Ideas of the projects were coming from high income countries… in low income countries we just become collectors and we would not be mentioned in articles… We put a standing rule...No research will be published in Africa without having an African contemporary as a partner.” [SSA senior male researcher] |
| language barrier/ Writing | “There is a language barrier to some extent… people have the perception that it all has to be in English and whenever it is in English then they don’t feel comfortable...” [MENA senior female researcher]  “We are not taught how to write…. I just have to say it… I think to have Americans or white people looking at it, they have to be polite because it can be a very sensitive thing for them to say anything. Our writing is atrocious.” [SSA senior female researcher] |
| Mentorship/ sponsorship/next gen | ” …mentorship, mentorship-attitude, mentorship-growth. encouraging people to truly mentor and build the next generation… requires a little bit of selflessness… you have to be willing to allow someone else to overtake you... How do we grow that culture, In a world where everybody wants to be top dog.” [SSA senior female researcher]  “Structured mentoring is very important where you have senior people mentoring the students then you are also having peer to peer mentoring among the students and the post-docs. So mentoring is going top-down but also bottom up and sideways, that’s very important.” [SSA senior female researcher]  “Researcher is really an apprenticeship, its learning by apprenticeship, which is mentorship.” [SSA senior male researcher] |
| Opportunities | “as part of this building, the ecosystem or the scientific community, they even give funding for high school students to do research, opportunities for internships. They go to schools sometimes and talk about research.” [MENA senior male researcher]  “Unfortunately, in the region there aren’t really attractive opportunities. It’s hard to find good opportunities especially when you compare resources. It was actually a struggle to find such an opportunity.” [MENA senior male researcher] |
| Publishing | “So I have written in Lancet that we have to support our national journals because if we send our papers to national journals then they will readily accept it, welcome it and publish it… instead of running after fancy journals who are very snobbish and who are going to refuse our papers. In the world of social media, it doesn’t matter where the paper is published, once you have the DOI, you can share it on email, on twitter, on face book… It will be known to everybody that you have published this paper… it doesn't have to be New England Journal of Medicine, American Journal of Surgery, Lancet or JAMA.” [ASIA senior male researcher] |
| Research experience | “I had a really good training in different science cultures and of course part of the culture here… I think it’s really important to get this training because people get exposed to a different way of thinking, to have things really done in an established scientific program and then when they come back, they come back with that experience to use here.” [MENA senior male researcher] |
| Research for research sake | “Students reached out to me… interested in getting our internship, from hospitals and colleges based in Bahrain… the students don’t want to research… they want the benefit of research that will get attached on their C.V when they are applying to the U.S.A or UK or Europe.” [ASIA senior male researcher] |
| Training & excellence | “So how do you connect what is in your head with your heart for good. I think training... I am a living blue print of a star. It’s not that......I am not making it up... I was trained as an undergraduate to do research and that I articulated to what I did in PhD.” [SSA senior female researcher]  “We are not training people in how to communicate and disseminate research especially in the written form. Oral is all very good and its excellent… but a lot of what goes to really change the research world needs to be written, needs to be published, needs to be in this peer reviewed format.” [SSA senior female researcher] |
| Trends and re-learning | “Nairobi University sits on their laurels that they are the best. Moi University came from being an underdog to trying to see what they could do, they were open to collaborations, they were open to more, they were open to opportunities … Moi definitely has given Nairobi a run for its money.” [SSA senior female researcher] |

| **RECOGNITION** | **Quotes** |
| --- | --- |
| Self-recognition (confidence/ imposter syndrome) | “We have limitations, we have historical issues but you still have to learn and the thing is, our brains are just as set up as wired as anybody else. We have to get our minds to do the work.” [SSA senior female researcher]  “It is really more about the doubts that we have in ourselves you know, because you have been trained...some people will think like I am not cut out for this, I am not good enough, so it’s real that lack of self-efficacy. lack of belief. I think it is really overcoming **that internalized goliath** we should start early... We have to make people know that you have something to contribute… that can make a difference.” [SSA senior male researcher] |
| Inequity and bias (epistemic injustice) | “when I joined university, it was still very much patriarchal and like very condescending and don’t see it disappeared because there are still some people who sort of say,” you won’t be able to do that, it’s kind of impossible"  So, I have taken it the other way round, every time somebody tells me you can’t do it, it’s impossible, I am like, “I am determined to show you that you are wrong,” [SSA senior female researcher]  “When I come home, I can do some research work or play with my children but for a woman, the same responsibilities plus running the family…, it is not taken very serious by institutions when a woman is asking for research funding and a man is different. It will be a burden on the woman” [SSA senior male researcher]  “Journals reject and are suspicious of African articles [paraphrased]. If I just send an article to the Lancet from Ethiopia and say your ”African name.” They will say who is “African name”? It’s very difficult, but if its someone like “John Miller” sending an article from Harvard sending, I don’t think it will have the same acceptance.” [SSA senior male researcher]  “Funds are there but you know, funds are...should I speak really? Funds are given to their favorites. There is a lot of favoritism… Every nation has crafty institutes, Central institutes where important people go for their treatment. The funding goes to these big institutions because they are in the funding committee of all the government departments…, it hardly ever comes to grassroots, never.” [ASIA senior male researcher] |
| Breaking glass ceilings | “But you have to break the glass ceiling. They will not offer it for you, they will not offer it for me. The global note, we have to break many glass ceilings and that is just one of them. Nobody is going to say, “Come on, welcome" Nobody is going to put a red carpet for you, come, you are welcome. You have to break each and every glass ceiling, but they will never talk about it, they will always say, “we are promoting women,” but it is not going to happen.” [ASIA senior male researcher] |
| Success definition | “if I stayed in the USA I would not have had the same success as I have it here, it’s definitely way more and the reason for this is because the resources here are better, in the USA, it is extremely competitive you know like getting a grant, it’s very competitive right now but here the competition is definitely less. So this allowed me to build much bigger things, to do lots of things yah. So, I have been very, very productive.” [MENA senior male researcher]  “Of course I can talk of the papers, at personal level these things really give a lot of contentment.  I mean just to see a whole generation you know, coming through, getting trained, and now they are actively working in many different places and they are active researchers and they are generating papers.” [MENA senior male researcher]  “There needs to be high profiled status within publication groups and not like middle authorship… So for me it’s about the respect and then and the recognition of the partners in those groups.” [SSA senior male researcher] |
| Communication & Public relations | “PR for research is not good. We do not do enough story telling about our research… even lay people do not know what you are doing, so there is just not enough PR on research to let it garner the economics needed to do the work. Students do not know the value…, in the community people don’t understand it, politicians don’t even know it… You know when McDonald is coming to town; it has good PR… with beautiful colors that make people get attracted to. Beer, tobacco, all have got good PR, they call in what you want, they pick it up and people use it. Research has no PR.” [SSA senior female researcher] |
|  | |
| **COLLABORATION** | ”It’s also about finding the right collaborators, building those personal relationships with the funding agents… what makes you smart is building those relationships with NIH grant program officers, the Gates foundation with the EU, the Canadians, those interpersonal relationships and networks… that’s where you hear information and things you have not seen published online.” [SSA senior male researcher]  “As part of this model, they contract several American universities to open campuses here and yah I got recruited fortunately in one of those universities called world Cornell medical college, which is part of the Cornel university in the USA." [MENA senior male researcher]  “Nature has a deal with the Egyptian government represented in the EKB, Egyptian Knowledge Bank, where they give classes to university staff and researchers to learn how better to write papers, what are the criteria, how to edit the papers and how the reviewers see it.” [MENA junior female researcher] |
|  | |
| **CULTURE** | “I think I would put some of that money in changing behaviors and mental model of potential young researchers, I am not giving up on the old ones but I think I would go to the ones that are still coming up. I will put significant resources in engaging the early, just as they are choosing careers to be letting them know where research is place, whether you are doing medicine, public health, nursing even social sciences, similar thing.” [SSA senior male researcher]  ”You need to surround yourself with the people who understand. It might seem like it is work to you, but this is life and death for some of us. And so I want to be around people who value that, who appreciate that, it is not about 8-5, it is about life and death.” [SSA senior female researcher] |
